# Pharmacological memory modulation to augment trauma-focused psychotherapy for PTSD: a systematic review of randomised controlled trials

**DOI:** 10.1038/s41398-023-02495-2

**Published:** 2023-06-15

**Authors:** Laura Meister, Ana Catarina Dietrich, Mina Stefanovic, Francesco Bavato, Alex Rosi-Andersen, Judith Rohde, Benjamin Offenhammer, Erich Seifritz, Ingo Schäfer, Thomas Ehring, Jürgen Barth, Birgit Kleim

**Affiliations:** 1 Department of Psychology, University of Zurich, Zurich, Switzerland; 2 Department of Psychiatry, Psychotherapy and Psychosomatics, Psychiatric University Hospital Zurich, Zurich, Switzerland; 3 Department of Psychology, LMU Munich, Munich, Germany; 4 Chronobiology and Sleep Research Group, Institute of Pharmacology and Toxicology, University of Zurich, Zurich, Switzerland; 5 Department of Psychiatry and Psychotherapy, University Medical Center Hamburg-Eppendorf, Hamburg, Germany; 6 Institute for Complementary and Integrative Medicine, University Hospital Zurich and University of Zurich, Zurich, Switzerland; 7 Neuroscience Center Zurich, University of Zurich, Zurich, Switzerland

**Keywords:** Diseases, Psychiatric disorders

## Abstract

Trauma-focused psychotherapy (tf-PT) is the first-line treatment for posttraumatic stress disorder (PTSD). Tf-PT focuses on processing and modulating trauma memories. Not all patients benefit, however, and there is room for improvement of efficacy. Pharmacologically augmenting trauma memory modulation in the context of tf-PT may help optimise treatment outcome. To systematically review effects of pharmacologically augmented memory modulation in the context of tf-PT for PTSD (PROSPERO preregistration ID: CRD42021230623). We conducted a systematic review of randomised controlled trials of psychotherapy treatment for PTSD. We included placebo-controlled studies that augmented at least one treatment session pharmacologically targeting memory extinction or reconsolidation. We calculated post-treatment between group (pharmacological augmentation vs placebo control) effect sizes of PTSD symptom severity. We included 13 RCTs. There was large heterogeneity in augmentation procedure and methodological quality. Four studies showed significantly greater PTSD symptom reduction in the pharmacological augmentation group (propranolol, hydrocortisone, dexamethasone, D-cycloserine) compared to placebo. Seven studies showed no significant effect of pharmacological augmentation compared to placebo (D-cycloserine, rapamycin, mifepristone, propranolol, mifepristone combined with D-cycloserine, methylene blue). Two studies showed significantly smaller PTSD symptom reduction in the pharmacological augmentation group (D-cycloserine, dexamethasone) compared to placebo. Results of pharmacological augmentation were mixed overall and heterogenous for the pharmacological agents tested in more than one study. Additional studies and replications are needed to identify which pharmacological agents work, in which combination and to identify patient groups that benefit most to tailor PTSD treatment.

## Introduction

Trauma-focused psychotherapy (tf-PT) is an efficacious and guideline-recommended, first-line treatment for posttraumatic stress disorder (PTSD) [1]. The therapeutic approach focuses on memory modulation via emotional and cognitive processing of the trauma [2, 3]. Patients recall and relive traumatic experiences and are confronted with trauma-related stimuli and reminders in the safe psychotherapeutic environment and under therapists’ guidance [2]. Unfortunately, not all patients benefit from this type of treatment and ~50% of patients report residual symptoms after treatment [4]. Hence, there is significant need and room for improvement of treatment effects. There are currently few approved pharmacotherapies for PTSD and they show only small effect sizes [5]. One possible route for improvement is to augment key tf-PT processes, such as memory modulation, by pharmacological agents. In such an approach, individual and specific tf-PT sessions are pharmacologically augmented in order to modulate key processes or mechanisms, rather than administering the medication over longer time [6].

In the context of tf-PT, pharmacological substances could augment memory modulation in the context of therapy sessions. Fear memory extinction and memory reconsolidation are critical components of tf-PT and may pharmacologically be augmented to optimise tf-PT. A number of pharmacological agents have been repurposed to act on the memory during psychotherapy, i.e., by strengthening or weakening memory extinction and reconsolidation, both in animal and human models [7–10]. Such basic science findings could be translated to the clinic to augment memory-based psychotherapeutic approaches, such as tf-PT, by strengthening extinction learning or reconsolidation blockade in the context of trauma memories [10]. A number of recent studies have indeed examined such pharmacological augmentation of tf-PT, but efficacy of these augmentation approaches has not yet been systematically evaluated [6, 11]. Since a number of studies have recently been published, the time is now ripe for a systematic review of the efficacy of pharmacological augmented interventions targeting memory extinction or reconsolidation.

Here we systematically review all randomised controlled trials investigating augmentation of tf-PT with pharmacological agents targeting trauma memory modulation, i.e., extinction or trauma memory reconsolidation. We aim to summarise findings on (1) the efficacy of tf-PT augmentation (2), the context under which such augmentation procedures deem successful, i.e, dose and timing of drug administration, content and length of augmented tf-PT sessions and (3) and side effects.

## Methods

We followed the Preferred Reporting Items for Systematic Reviews and Meta-Analyses (PRISMA) statement for conducting and reporting of this meta-analysis [12] (see Supplementary Appendix B, Table 1). The review protocol was prospectively registered with the International Prospective Register of Systematic Reviews (registration number CRD42021230623 [3]).

### Eligibility criteria

We identified all English-language randomised controlled trials (RCTs) published in peer-reviewed journals that comprised 1) trauma-focused psychological treatment for PTSD; 2) adult participants (≥18 years old) 3), 3) at least 70% of participants diagnosed with PTSD (i.e., according to DSM-IV/V, ICD-10); 4) PTSD severity as primary outcome; 5) at least ten participants per treatment group; 6) pharmacological augmentation of at least one treatment session to target memory modulation (reconsolidation or extinction); and 7) comprised a placebo control group for the pharmacological intervention. We included RCTs based on any type of trauma, type of setting (i.e., inpatients, outpatients), comorbidity, or additional medication use.

### Study selection

A systematic literature search was conducted for full articles on the databases PubMed, PsycINFO, PSYINDEX, PTSDpubs and Cochrane Library until February 28, 2021. The search strategy included the following keywords:(PTSD OR posttraumatic stress disorder OR posttraumatic stress disorder OR PTSD) AND ((treatment trial OR randomised controlled trial OR clinical trial) OR (*indexed by a thesaurus term as clinical trial*)). Search terms had to be found in the title or abstract. No other limitations were used. The search was originally performed in preparation for the German S3 treatment guidelines for PTSD [1] and covered all publications until May 2015. The search was updated in four additional waves using identical criteria and interventions covering all publications until 28 February 2021 (Wave 2: May 2015 - May 2017; Wave 3: May 2017 - August 2019; Wave 4: September 2019 - May 2020; Wave 5. June 2020 - February 2021).

In addition, we performed a systematic snowball search by screening reference lists from included primary studies and relevant review articles. In the second step, the remaining articles were further screened for pharmacological augmentation eligibility criteria based on title and abstract. We selected 50 studies from the 620 RCT’s of the updated S3 database for full text screening. We included 13 studies that included pharmacological augmentation and fulfilled the above-mentioned eligibility criteria

**Fig. 1:**
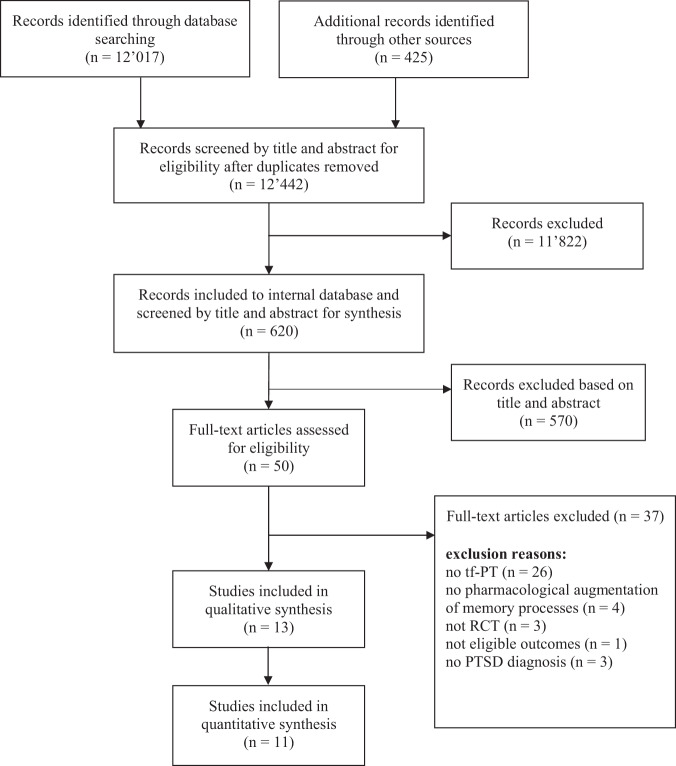
Study flow (PRISMA). Note. RCT randomised controlled trial, FU follow-up.

Two researchers independently screened articles and decided on eligibility of the study. In cases of disagreement between the researchers, a third researcher decided on eligibility. The selection of studies is depicted in the PRISMA flowchart (see Fig. 1).

### Data extraction

The following study characteristics were extracted: Publication year, authors, sample size, sex, mean age, PTSD diagnosis, trauma type, study origin, study design, PTSD symptom assessments, including measurement times, follow-ups and intervention type. Type of pharmacological intervention was categorised according to the proposed working mechanism declared by the authors of the original studies, i.e., pharmacological augmentation of fear extinction or impairing trauma memory reconsolidation. With respect to trauma type, a study was categorised to one of five trauma types (physical assault, sexual assault, accident (including road traffic accident), natural disaster, combat, mixed) when the majority (70%) of participants had experienced this trauma type.

### Outcome measures

Primary outcome was post-treatment PTSD symptom severity. We used the first post-treatment assessment after the last intervention session to calculate effect sizes related to PTSD symptom severity in the pharmacologically augmented group vs. the placebo group. For all studies, results from intention-to-treat (ITT) analyses were used, if available. Where no ITT analysis was conducted, results from per-protocol analyses were used instead. When a study used self-report and clinician administered measures for PTSD outcome, both measures were combined and averaged to obtain a more informative outcome measurement (see Supplementary Appendix A) [13]. The same was done when studies used several measures for self-reported or clinician administered PTSD symptoms. Similar to this, subscale scores were also pooled using the same formula when a study reported only scores for the subscales of a measure but not a total score (see Supplementary Appendix A).

### Study quality assessment

Risk of bias was assessed with the Cochrane Risk of Bias Tool [14]. The following five domains were assessed: (1) bias arising from the randomisation process (2), bias due to deviations from intended interventions (3), bias due to missing outcome data (4), bias in measurement of the outcome and (5) bias in selection of the reported result. Risk of bias can be rated as *low*, *some concerns* or *high risk*. For each domain, we rated the risk. The answer option *no information* was used when the published paper did not provide enough information to fully answer the signalling question. When any of the domains within a study were rated *high risk* or more than two domains were rated *some concerns*, the study was regarded overall at high risk for any bias. Two researchers independently coded risk of biases and consulted a third in case of disagreement.

### Data analysis

Effect sizes were estimated with Hedges’ g [15]. For calculation of effect sizes, means, standard deviations (SD) and sample size per treatment group were extracted. Where these statistical values were not reported, other values such as standard error (SE) or confidence interval (CI) were used to replace them or for their calculation respectively. When a study did not contain enough information for data extraction or calculation, study authors were contacted. Effect sizes were obtained for pairwise comparisons of post-treatment. The studies were weighted by sample size using the inverse-variance technique. All statistical analyses were conducted with the *metafor* package in *R* (version 1.2.5001) [16].

To test publication bias we applied Egger’s regression test [17]. There was no significant Egger’s regression test (*p* = 0.9).

## Results

### Included studies

**Fig. 2:**
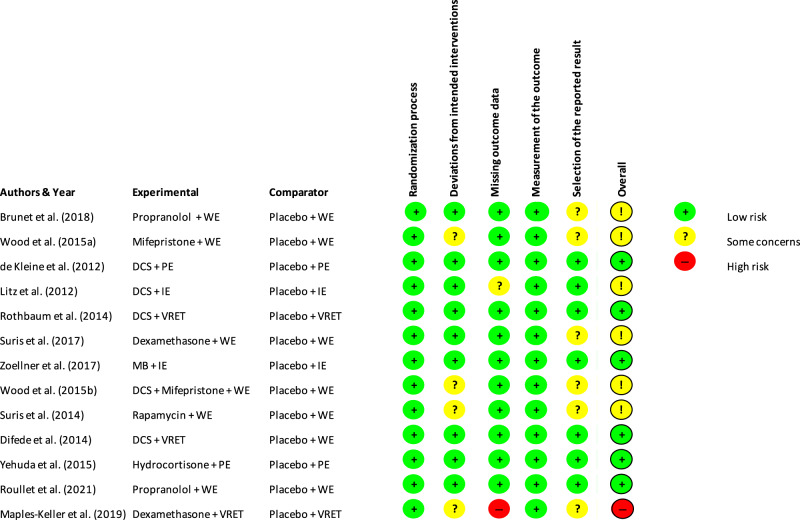
Risk of bias assessment. Note. For abbreviations see Supplementary Appendix B, Table 2.

We included thirteen studies (total *N* = 583, range 24–156) (see Fig. 1). Table 2 presents an overview of selected studies characteristics, including sample characteristics (sample size, sex, mean age and trauma type), pharmacological intervention, proposed mechanism, characteristics of the tf-PT protocol, homework, augmented tf-PT (total sessions and number of augmented sessions), augmentation regime, side effects, PTSD assessment, follow-up assessments and significant group per time PTSD symptom improvement. Studies were mainly carried out in the USA (*n* = 11). Two studies were carried out in Canada (*n* = 1) and France (*n* = 1). Studies used pharmacological agents that targeted trauma memory extinction (*n* = 8) or reconsolidation (*n* = 5) (see Supplementary Appendix B, Table 3 for a summary of the agents and a description of their pharmacological working mechanism). The selected studies used seven different pharmacological agents: D-cycloserine (DCS) (*n* = 5), hydrocortisone (HC) (*n* = 1), propranolol (*n* = 2), rapamycin (n = 1), dexamethasone (DEX) (*n* = 2), mifepristone (*n* = 2) and methylene blue (MB) (*n* = 1). Patient samples reported combat-related trauma (*n* = 7), war experience as civilians (*n* = 1) and mixed trauma (*n* = 5). Across studies, the mean number of augmented sessions was 5.54 (Md = 5) sessions, ranging from one to 11 sessions. Trauma memory exposure took place either within therapy sessions based on prolonged exposure therapy (PE), imaginal exposure (IE), virtual reality exposure therapy (VRET) or through written narrative exposure (WE). In the chapter *pharmacological augmentation effects*, we summarised the study protocol and findings of each individual study sorted by pharmacological agents. Effect sizes of individual studies are depicted in Fig. 2.

### Risk of bias assessment

**Fig. 3:**
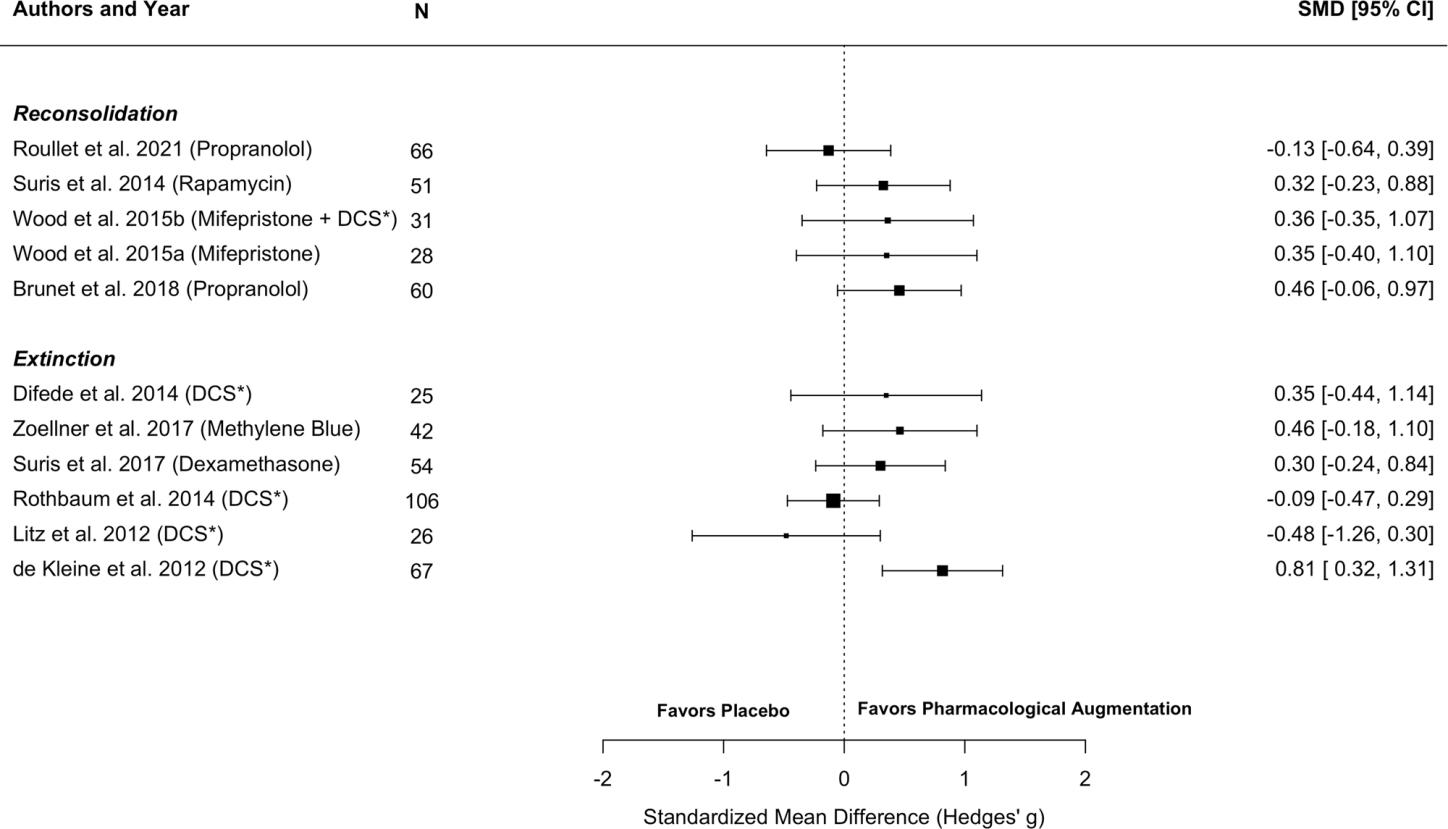
Effect sizes of the included studies. Note. Effect sizes and confidence intervals based on individual study results and grouped by reconsolidation and extinction as target for augmentation. Boxes reflect effect sizes of the individual study. Box size is weighted according to sample size of the studies. *DCS = D-cycloserine. Due to the large heterogeneity in pharmacological agents, memory modulation target and session content, overall effects are not calculated.

Risk of bias assessment yielded six studies rated at low risk, six studies with some concerns and one study rated at high risk of bias (see Fig. 3). Concerns were mainly rated in the domains *deviations from intended interventions* and *selection of reported result*. For the latter, this related to missing information on a pre-specified analysis plan unrelated to the current analysis.

### Pharmacological augmentation effects

#### D-cycloserine

De Kleine et al. (2012) augmented 10 sessions of PE with a mean duration of 30 min per session, with 50 mg DCS in 67 patients with PTSD following mixed trauma [18]. During the augmented sessions, patients were asked to vividly recall the traumatic event with sensory details and as vividly as possible for 30 min. Between sessions, patients listened to audiotapes of their exposure sessions five times a week and engaged in vivo exposure to trauma-related stimuli from the third session onward. No significant differences in PTSD symptom improvement were observed between the DCS and the placebo group. DCS was associated with a marginally greater PTSD symptom improvement compared to placebo. Significantly stronger effects of DCS were observed in patients with higher PTSD symptom severity and for who patients that did not achieve 70% PTSD symptom reduction within the first seven sessions (see Table 2 for effect sizes (Hedges’ g) for all selected studies).

Difede et al. (2014) augmented 10 sessions of VRET with a mean duration of 90 min per session, with 100 mg DCS in 25 patients with PTSD following civilian trauma (World Trade Center terrorist attack (WTC)) [19]. During the sessions, patients were asked to vividly recall their traumatic experience for 45 min with as many sensory details as possible. Imaginal exposure was enhanced with a computer simulation of the 11^th^ September WTC attacks. DCS was associated with a significantly greater PTSD symptom improvement compared to placebo, with differences commencing post session 6 with maintained effects at 6-month follow-up. Additionally, sleep was significantly improved in the DCS group compared to the placebo group. Stronger effects in the DCS group were observed in patients with no concurrent medication compared to those with stable medication.

Litz et al. (2012) augmented 4 sessions of IE with a mean duration of 90 min per session, with 50 mg DCS in 26 male patients with PTSD following combat trauma [20]. During the sessions, patients were asked to vividly recall their most distressing war memory for 45 min. This was followed by processing and discussing the memory for 10 min with the therapist. Contrary to the hypotheses, DCS was associated with significantly poorer PTSD symptom improvement compared to placebo. Patients in the DCS group rated an increase in subjective units of distress scale (SUDS) from the first to the second session whereas patients in the placebo group reported a decrease.

Rothbaum et al. (2014) augmented 5 sessions of VRET with a mean duration of 90 min per session, with 50 mg DCS or 0.25 mg alprazolam in 156 patients with PTSD following combat trauma [21]. For the present analysis, we focused on DCS/placebo. During the sessions, patients were encouraged to expose themselves to their most traumatic memories for around 30–45 min. Imaginal exposure was virtually augmented with trauma related stimuli that were matched to the patient’s narrative. This was followed by processing and discussing the memory with the therapist for 15–20 min. No significant differences in PTSD symptom improvement were observed between the DCS and the placebo group. Significantly stronger effects of DCS were found in patients with more session-to-session learning (assessed by the average decrease in peak ratings of subjective discomfort across exposure sessions). Additionally, DCS was associated with greater posttreatment reductions in cortisol and startle reactions than placebo. The authors did not provide information on adverse events.

#### Glucocorticoids

Suris et al. (2017) augmented 4 sessions (1) of WE (unknown duration) and (2–4) 30–40-s script-driven imagery with 0.15 mg/kg (12 mg/80 kg) long-acting DEX in 54 PTSD patients following combat trauma [22]. In the first session, patients provided a written narrative of two traumatic events and identified bodily responses they had experienced during trauma. The trauma narratives were then edited, and audio recorded into two separate narratives by study staff with insertion into the narrative of up to five bodily responses. In sessions 2–4 during script-driven imagery, patients were then exposed to the two traumatic recordings of 30–45 s of duration as well as to two neutral recordings. DEX was associated with a significantly greater PTSD symptom improvement compared to placebo. One serious adverse event was reported in the DEX group which led to hospitalisation and rated as possibly related to the study.

Maples-Keller et al. (2019) augmented 11 sessions of VRET, with a mean duration of 90 min per session, with long-acting 0.5 mg DEX in 27 patients with PTSD following combat trauma [23]. During the sessions, patients were asked to vividly recall their trauma memory for 30–45 min. Imaginal exposure was virtually augmented with visual and audio cues of military scenarios, followed by processing and discussing the memory for 20 min. DEX was associated with a significantly poorer PTSD symptom improvement compared to placebo. Due to the high drop-out rate >50% (77% in the DEX group vs. 28.5% in the placebo group) unblinding was performed earlier and further recruitment stopped prematurely.

Yehuda et al. (2015) augmented 8 sessions of PE (unknown duration), with 30 mg short-acting HC (equivalent to 1.12 mg long-acting dexamethasone) [24] in 24 patients with PTSD following mixed trauma [25]. HC was associated with a significantly greater PTSD symptom improvement compared to placebo. However, when comparing post-treatment symptoms without including initial symptom severity, the group difference was nonsignificant. There was a higher drop-out rate in the placebo group (58%) compared to the HC group (8%). Significantly stronger effects for HC were observed in patients with higher PTSD symptom severity.

#### Methylene blue

Zoellner et al. (2017) augmented 5 sessions of IE with a mean duration of 50 min per session, with 260 mg MB in 42 patients with PTSD following mixed trauma [26]. Patients were randomised to an intervention group and two different control conditions (placebo, waitlist). For the present analysis, we focused on MB/placebo. During the sessions, patients were asked to vividly recall their traumatic event for 30–45 min. This was followed by processing and discussing the memory with a therapist for 15 min. No significant differences in PTSD symptom improvement were observed between the MB and the placebo group. Results from session-to-session analyses of PTSD symptom severity showed a delayed and later accelerated clinical gains over the entire course of 5 sessions in comparison to placebo. Strongest effects of MB were observed in patients with better working memory capacity.

### Pharmacologically targeting reconsolidation

#### D-cycloserine

Wood et al. (2015b) augmented one session of WE (unknown duration) with 100 mg DCS and 180 mg mifepristone in 31 patients with PTSD following mixed trauma [27]. In this session patients had to write scripts including details of two traumatic experiences, which had led to patients’ PTSD, and of nontraumatic experiences. One week later, patients were engaged in script-driven traumatic mental imagery for 30–40 s. No significant differences in PTSD symptom improvement were observed between the DCS/Mifepristone and the placebo group.

#### Propranolol

Brunet et al. (2018) augmented 6 sessions of WE, with a mean duration of 10–20 min, with 0.67 mg/kg (53.6 mg/80 kg) short-acting +1 mg/kg (80 mg/80 kg) long-acting propranolol in 60 patients with PTSD following mixed trauma [28]. In the first session, patients were instructed to write down a personal trauma narrative focusing on the most disturbing moments, in present tense and first-person singular, including five or more bodily sensations drawn from a checklist (up to 30 min). During the five following weekly sessions patients were asked to read this trauma narrative aloud to the therapist “as if they were back in the event” which took about 10–20 min. Propranolol was associated with a significantly greater PTSD symptom improvement compared to placebo at one-week FU.

Roullet et al. (2021) augmented 6 sessions of WE with a mean duration of 3–10 min, with 0.67 mg/kg (53.6 mg/80 kg) short-acting +1 mg/kg (80 mg/80 kg) long-acting propranolol in 66 patients with PTSD following mixed trauma [29]. Patients were instructed to write a trauma narrative containing the most disturbing moments of their trauma for up to 30 min. In the following sessions, patients were asked to read their narrative aloud to the therapist in order to reactivate their trauma memories for 3–10 min. No significant differences in PTSD symptom improvement were observed between the propranolol and the placebo group at 1-week FU. After three months, in patients with higher PTSD symptom severity (PCL-S > 65) symptoms continued to decline in the propranolol group but increased in the placebo group. Two patients reported adverse events in the propranolol group and non were reported in the placebo group.

#### Rapamycin

Suris et al. (2014) augmented one session of WE with a mean duration of 45 min with 15 mg rapamycin in 54 male patients with PTSD following combat trauma [30]. The task consisted of preparing a script containing the most distressing parts of the combat trauma as well as five bodily sensations experienced during trauma. One week later, patients were exposed to script-driven imagery for 30–40 s. No significant differences in PTSD symptom improvement were observed between the rapamycin and the placebo group. Marginally greater PTSD symptom improvement was observed in the rapamycin group compared to the placebo group. Stronger effects of rapamycin were observed in patients with less chronic PTSD. They study authors reported that WE for some participants took place outside of the rapamycin peak levels.

#### Mifepristone

Wood et al. (2015a) augmented one session of a WE (unknown duration) with 30 mg/kg (2400 mg/80 kg) mifepristone in 43 patients with PTSD following mixed trauma [27]. Patients were instructed to write scripts including details of traumatic experiences, which lead to their PTSD, as well as of three nontraumatic experiences. One week later the patients were engaged in script-driven imagery for 30–40 s. No significant differences in PTSD symptom improvement were observed between the mifepristone and the placebo group.

### Publication bias

**Fig. 4:**
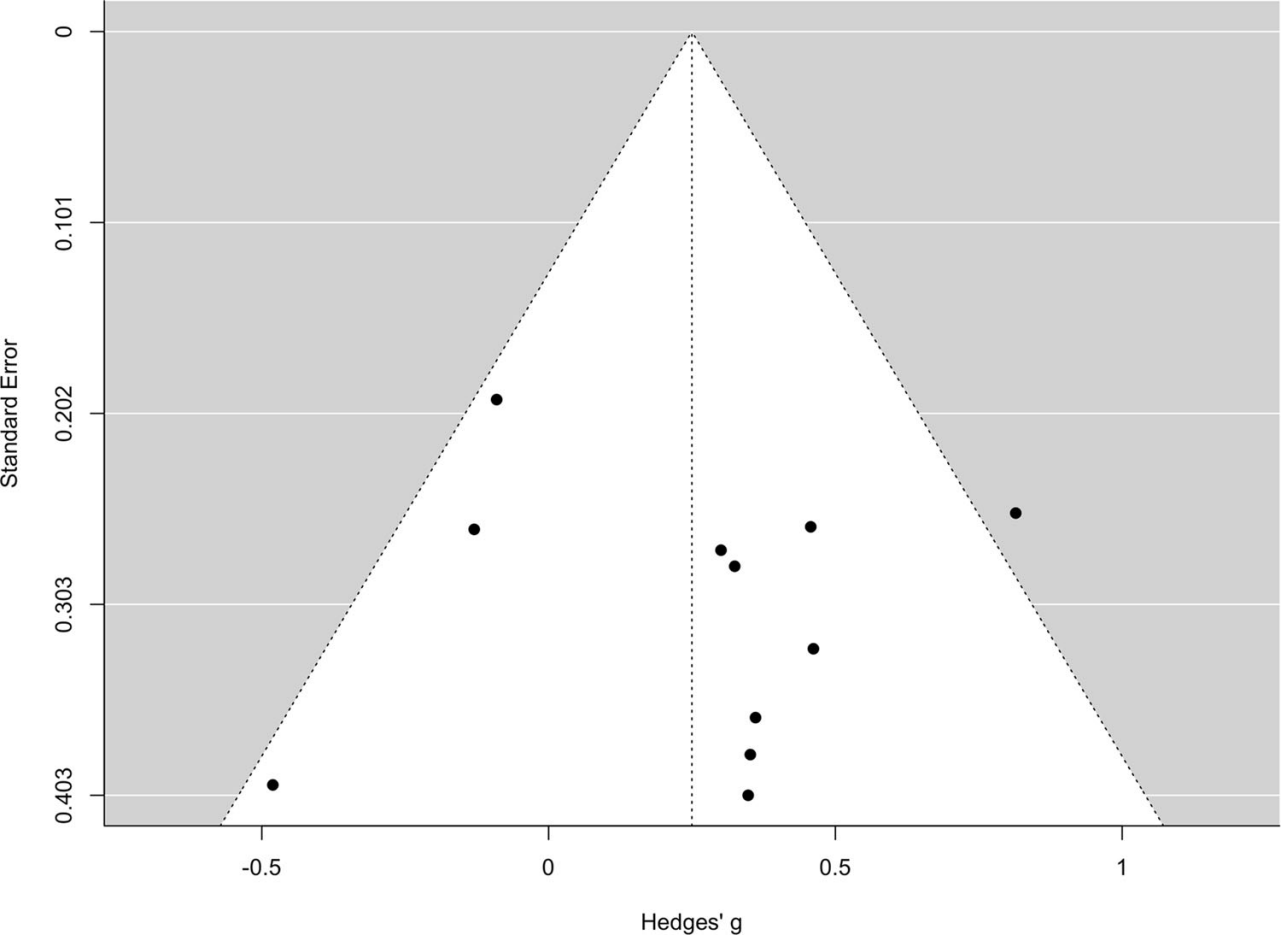
Funnel plot. Note. Funnel plot for the pharmacologically augmented intervention studies. No asymmetry was detected (*k* = 11, *intercept (B0)* = 0.23, *95%CI*[−0.82, 1.28], *p* = 0.97).

We assessed potential publication bias via funnel plot asymmetry and performed an Egger’s test to test for significant deviations. There was no significant asymmetry in the funnel plot detected *(k* = 11*, intercept (B0)* = 0.23*, 95% CI*[−0.82, 1.28]*, p* = .97*)* (see Fig. 4).

## Discussion

We included 13 randomised-controlled PTSD treatment trials that pharmacologically augmented memory modulation in at least one session of trauma-focused psychotherapy. The pharmacological agents used to augment treatment sessions were DCS, DEX, HC, propranolol, mifepristone, rapamycin and combination of DCS and mifepristone. Despite the increasing interest in psychopharmacological augmentation as a strategy to improve treatment outcome in psychotherapy for PTSD treatment, none of the reported augmentation strategies have been officially approved by regulatory bodies. The strategies are thus to be considered off-label or experimental and their efficacy and safety should be carefully evaluated, which was the aim of this work to contribute to. Overall, there was considerable heterogeneity across studies in how tf-PT was delivered and pharmacologically augmented, varying from one to 12 weekly sessions with session duration from several minutes to 90 min and augmentation of one to 11 sessions [18, 29]. Some, but not all treatments comprised between-session homework, e.g., listening to the trauma narrative or practicing in vivo exposure. Three agents, DCS, DEX and propranolol, were used in more than one study. Four studies investigated DCS, with expected effects for extinction augmentation. DCS augmentation was associated with significantly greater PTSD symptom improvement compared to placebo in one study [19], while it was associated with a significantly smaller PTSD symptom improvement in another [20]. Two studies reported no significant difference between the DCS and placebo [18, 21]. The studies thus reported mixed findings, but they also differed significantly regarding patient populations (veterans, vs. civilian patient samples), treatment protocol (Virtual Reality Therapy vs. Imaginal Exposure), number and duration of sessions (4–10, 30–90-min sessions augmented) and DCS dosage (50–100 mg) [18–21]. Dose and timing of DCS may have accounted for some of the heterogenous results and future replication studies are warranted. Two studies investigated DEX, with expected effects for extinction augmentation. In one study, DEX augmentation was associated with greater PTSD symptom improvement [22], while in another study it was associated with a smaller PTSD symptom improvement [23]. The studies differed significantly regarding the treatment protocol (Written Exposure vs. Virtual Reality Therapy), number of treatment sessions (4 vs. 12), in dosage (12 mg vs 0.5 mg) and timing of DEX (60 min vs. 773 min prior to tf-PT) which may account for the heterogenous results.

Two studies investigated propranolol, with expected effects for reconsolidation blockade. Pharmacological augmentation was associated with significantly greater PTSD symptom improvement compared to placebo in one study [28] but there was no association in the other study [29] even though in this case both studies used similar treatment protocols.

Some patient samples may be more likely to respond to pharmacological augmentation. A sample consisting of survivors of the WTC attack [19], for instance, may show greater responses than mixed trauma or combat trauma samples due to higher trauma load, medication, comorbidity, chronicity and longer PTSD symptom duration in the latter group [31]. Five studies reported associations between augmentation effects and concurrent medication, baseline PTSD severity and chronicity. In one study, patients with higher baseline PTSD symptom severity were most likely to respond to HC augmentation [32]. Such an association was also observed in two studies that did not report an effect of propranolol [29] and DCS augmentation [18] in the overall group. However, a study without overall rapamycin effects reported that the subgroup with less chronic PTSD showed greater symptom improvement compared to those with more chronic PTSD under rapamycin augmentation. This accords with preclinical findings that recent memories are more amenable to pharmacological reconsolidation blockade than more remote chronic traumatic memories [31].

For pharmacological agents used to augment extinction, extinction and/or safety learning must occur in the respective augmented sessions in order to successfully enhance and detect such effects. DCS enhanced VRET effects in patients that showed extinction learning during VRET, which was indexed by decrease in subjective distress during exposure, but not in patients without such decrease [21]. Such between-person response differences to tf-PT should thus be indexed alongside other moderating factors to derive clear implications. Furthermore, the findings from one study suggest that in the absence of extinction learning, DCS augmentation may be associated with smaller PTSD symptom reduction [20].The authors reason that in this case DCS could have led to enhanced consolidation of the stressful experience after the first tf-PT session rather than the augmentation of extinction learning. This was reflected in an increase of subjective distress from the first to the second session. One way to prevent these adverse effects is optimising timing of drug administration.

Timing of pharmacological augmentation plays a key role in successfully targeting desired memory processes. Zoellner (2017), for instance, highlighted that MB may delay initial gains of therapy by consolidating the stressful experiences in initial sessions rather than successfully augment extinction learning, which may only occur later in therapy. A key challenge may thus be to tailor psychotherapy session content and precise modulation procedures to augmentation to achieve maximum effects. Pharmacological augmentation may otherwise not be optimal and may disguise or counteract effects [30]. Drug administration occurred predominantly *prior* to tf-PT sessions, except for one study [26]. As some agents may also decrease fear response or act on other processes, such as memory retrieval, such effects may influence outcomes, for instance in one study where drug effects were associated with drop-out rates [23].

In terms of study quality overall, six studies were rated as low risk of bias, six as some and one as high risk. The overall low risk of bias may reflect the fact that we included randomised controlled trials in this study, which per se mostly fall into the lower risk of bias assessment categories. No publication bias could be detected. Most studies were conducted in the USA or in Canada, hence raising the question about the generalisability to populations of other nationalities.

A number of pharmacological agents have been found to successfully facilitate fear extinction or block reconsolidation in animal models [9, 33, 34]. Such findings have led to calls for translation to human models and PTSD patients. However, this has been challenging as some of the most potent pharmacological agents cannot be used in humans due to their toxicity [35, 36]. For those agents successfully translated to pharmacological human fear memory manipulation in healthy participants, findings from PTSD patients are mixed [7]. These heterogenous findings may reflect the fact that trauma memories in PTSD are complex and their modulation associated with a number of processes operating in parallel [10]. During psychotherapy, trauma memory extinction and reconsolidation might often occur hand in hand and this may need to be disentangled in future studies.

We envisage three ways forward in this area for clinical research. First, there is a need for larger RCTs. The reviewed studies show that subgroups (i.e., patients with more severe PTSD) might be differentially susceptible to pharmacological augmentation. A meta-analysis or meta-regression for mean dose or trial duration could not be conducted as the trials examined completely different substances. Large heterogeneity was detected, as they involved not only different substances with distinct properties of action, but also different potential side effects and different dosing regimens in the studies. It was therefore impossible to combine mean data from these trials. Additionally, the studies differed significantly in terms of their design, patient populations, and other factors that could have impacted outcomes. As a result, attempting to conduct a meta-analysis or meta-regression analysis could have led to inaccurate or misleading results due to confounding. Based on research in statistics [37] at least five homogenous studies would be necessary, as well as corrections, such as the Hartung and Knapp procedure when the number of studies is lower, to avoid a high probability of Type 1 error. From a personalised medicine perspective, investigations in larger samples discovering which type of augmentation works best for which patients could help tailor interventions and exact augmentation regimes to individual patients. Second, future studies should investigate within-session as well as session-to-session changes capture whether respective tf-PT sessions were indeed successful. Such sessions could then be exclusively augmented. Third, some studies did not report the exact timing and duration of the trauma memory reactivation and reactivation may have taken place outside the peak pharmacological concentration for some patients. Future studies will need to tailor their augmentation regimes precisely to the trauma memory changes in tf-PT and record as accurate as possible the changes in trauma memories [38–42].

Taken together, there is not yet enough evidence to conclude on positive effects for the pharmacological agents used to augment tf-PT. More evidence is required to understand the precise mechanisms and conditions (timing, duration, session number, subgroups) under which pharmacological augmentation of tf-PT renders successful, hence pointing to the need for more research to successfully implement pharmacological augmentation in clinical practice and augment treatment for PTSD patients.

## Supplementary information

PRISMA Checklist

Appendix Tables
