# Supplementary material for: Pharmacological memory modulation to augment trauma-focused psychotherapy for PTSD: a systematic review of randomised controlled trials
Source: Transl Psychiatry. 2023 Jun 15;13:207. doi: 10.1038/s41398-023-02495-2 (PMC10272208; doi:10.1038/s41398-023-02495-2)
Supplement: Supplementary file 2 — Appendix Tables [file 41398_2023_2495_MOESM2_ESM.docx]

**Appendix**

**Appendix A: Effect Sizes of Different Outcomes for Included Studies**

Formula for combining two outcome measures and / or two groups:

$$M_{combined} = \frac{N1M1 + N2M2}{N1 + N2}$$

$${SD}_{combined}=\sqrt{\frac{\left( N_{1}-1 \right){SD}_{1}^{2}+\left( N_{2}-1 \right){SD}_{2}^{2}+\frac{N_{1}N_{2}}{N_{1}{+N}_{2}}(M_{1}^{2}+M_{2}^{2}-2M_{1}M_{2})}{N_{1}+N_{2}-1}}$$

where Ni is the sample size Mi the mean and SDi the standard deviation of each group included for the analysis at this measurement time.

**Appendix B: Tables**

**Table 2. Characteristics of Pharmacologically Augmented Randomised Placebo-Controlled PTSD Treatment Trials**

| **Authors (Year)** | **Sample**  **characteristics** *(N♀:no, mean age, trauma type)* | **Pharmacological**  **agent** | **Proposed**  **Augmentation**  **Mechanism** | **tf-PT**  *maximal*  *session number,*  *PT* | **Home**  **work** | **Augmented  tf-PT**  ***Total***  ***Sessions(***  ***Augmented sessions)*** | **Augmentation**  **Regime** | **Side Effects** | **PTSD**  **Assessment** | **Follow up assessment** | **Significant Group x time effect on PTSD symptom improvement** |
| --- | --- | --- | --- | --- | --- | --- | --- | --- | --- | --- | --- |
| de Kleine et al. (2012) | N = 67 (♀: 58)  *M* = 38 y/o  Trauma: mixed | EG: D-cycloserine | Extinction | 10^1^ weekly 30-min sessions of PE | yes | 10 (1-10) | 50 mg DCS or placebo  60 min prior to sessions | no | CAPS  PSS-SR  M.I.N.I. | T2: 1-week FU  T3: 3-month FU | None |
| Difede et al. (2014) | N = 25 (♀: 6)  *M* = 45.8 y/o  Trauma: civilian war experience | EG: D-cycloserine | Extinction | 12 weekly 90-min sessions of VRET | yes | 10 (2-11) | 100 mg DCS or  placebo  90 min prior to  sessions | no | CAPS  PCL | T2: immediately post-treatment  T3: 6-month FU | DCS > Placebo  at T2 (CAPS) and  T3 (CAPS, PCL) |
| Litz et al. (2012) | N = 26 men  *M* = 32.2 y/o  Trauma: combat | EG: D-cycloserine | Extinction | 6 weekly 90-min sessions of IE | no | 4 (2-5) | 50 mg DCS or  placebo  30 min prior to  sessions | no | CAPS  PCL-M | T2: post-treatment  T3: 3-month FU  T4: 6-month FU | Placebo > DCS  At T2 (CAPS, PCL-M) |
| Rothbaum et al. (2014) | N = 156 (♀: 8)  *M* = 35 y/o  Trauma: combat | EG: D-cycloserine  CG 2: Alprazolam | Extinction | 6 weekly 90-min  sessions of VRET | no | 5 (2-6) | 50 mg DCS or 0.25  mg alprazolam  (active control) or  placebo  30 min prior to  sessions | UNK | CAPS  PSS-R | T2: post-treatment  T3: 3-month FU  T4: 6-month FU  T5: 12-month FU | None |
| Maples-Keller et al. (2019) | N = 27 men  *M* = 35.4 y/o  Trauma: combat | EG: Dexamethasone | Extinction | 12^2^ weekly 90-min session of VRET | UNK | 11 (2-12) | 0.5 mg DEX the night (772.52 min) prior to sessions | yes | CAPS  PSS | T2: post-treatment | Placebo > DEX (At T2 CAPS, PSS) |
| Surís et al. (2017) | N = 54 men  *M* = 37.5 y/o  Trauma: combat | EG: Dexamethasone | Extinction | 1 session of WE (unknown duration) and 3 weekly sessions of 30-45-sec script-driven imagery | no | 4 (1-4) | 0.15 mg/kg DEX  (12 mg/80 kg)  or placebo  60 min prior to  session | yes | PCL | T2: immediately post-treatment  T3: 1-month FU  T4: 3-month FU  T5: 6-month FU | DEX > placebo  at T3 and T4 |
| Yehuda et al. (2015) | N = 24 men  M = 49.6 y/o  Trauma: combat | EG: Hydrocortisone | Extinction | 10 weekly sessions of PE  (unknown duration) | UNK | 8 (3-10) | 30 mg long acting  HC (equivalent to  1.12 mg DEX)  or placebo  20 min prior to sessions | no | CAPS  PSS-SR | T2: immediately post-treatment  T3: 6-week FU | HC > Placebo ^3^  at T2 and T3 (CAPS) |
| Zoellner et al. (2017) | N = 42 (♀: 30)  M = 37.5 y/o  Trauma: mixed | EG: Methylene Blue  CG 2: Waitlist | Extinction | 6 daily 50-mins sessions of IE | no | 5 (2- 6) | 260 mg MB  or placebo  Immediately after sessions |  | PSS-I  PSS-SR  PTCI | T2: 2-week FU  T3: 1-month FU  T4: 3-month FU | None |
| Brunet et al. (2018) | N = 60 (♀: 35)  *M* = 39.4 y/o  Trauma: mixed | EG: Propranolol | Reconsolidation | 6 weekly sessions of (1) 30-min and (2-6) 10-20-min WE | no | 6 (1-6) | 0.67 mg/kg short-acting (53.6 mg/80 kg) + 1 mg/kg (80 mg/80 kg) long-acting propranolol or placebo 90 min prior to sessions | yes | CAPS  PCL-S | T2: 1-week FU  T3: 6-month FU | Propranolol > Placebo  at T2 (CAPS and PCL-S) |
| Roullet et al. (2021) | N = 66 (♀: 41)  *M* = 38.9 y/o  Trauma: mixed | EG: Propranolol | Reconsolidation | 6 weekly sessions of (1) 30-min and (2-6) 3-10-min WE | no | 6 (1-6) | 0.67 mg/kg short-acting (53.6 mg/80 kg) + 1 mg/kg (80 mg/80 kg) long-acting propranolol or placebo 90 min prior to sessions | yes | SCID PTSD module  PCL-S | T2: 1-week FU  T3: 3-month FU | None |
| Wood et al. (2015a) | N = 43 (♀: 10)  *M* = 43.87 y/o  Trauma: mixed | EG: Mifepristone | Reconsolidation | 1 session of WE  (unknown  duration) and  30-40-sec script-  Driven imagery  one week later | no | 1 (1) | 30 mg/ kg  Mifepristone (2400  mg/80 kg)  or placebo  90 min prior to  session | no | IES-R | T2: 1-week FU | None |
| Wood et al. (2015b) | N = 31 (♀: 17)  *M* = 38.61 y/o  Trauma: mixed | EG: Mifepristone  + DCS | Reconsolidation | 1 session WE  (unknown  duration) and  30-40-sec script-  driven imagery  one week later | no | 1 (1) | 1800 mg  Mifepristone  or placebo 90 min  + 100 mg DCS  360 min prior to  session | no | IES-R | T2: 1-week FU | None |
| Surís et al. (2014) | N = 54 men  *M* = 43 y/o  Trauma: combat | EG: Rapamycin | Reconsolidation | 1 session of 30-75-min WE and  30-40-sec script-driven imagery  one week later | no | 1 (1) | 15 mg Rapamycin  or placebo  immediately before session | no | CAPS  PCL | T2:1-month FU  T3: 3-month FU | None |

***Notes*:** *CAPS* = Clinician Administered PTSD Scale, *CG* = control group, *DCS* = D-cycloserine, *DEX* = Dexamethasone (0.75mg DEX equivalent to 20mg HC), *EG* = experimental group, , *FU* = follow-up*, HC = Hydrocortisone (20mg HC equivalent to 0.75mg DEX), IES-R* = Impact of Event Scale Revised*, PCL* = PTSD Checklist, *PCL-M* = PTSD Checklist – Military Version, *PCL-S* = PTSD Checklist – Specific, *PSS-SR* = PTSD Symptom Scale – Self Report, *PCL* = PTSD Checklist, *IE* = imaginal exposure, *IES-R* = Impact of Event Scale Revised, *MB* = Methylene blue, *PE* = Prolonged Exposure, *PSS-I* = PTSD Symptom Scale – Interview, *T1-T4* = time point 1-4, *VRET* = Virtual Reality Exposure Therapy. *WE =* Written narrative exposure*. Wood et al. (2015)* contained three independent studies of which two (study 2 and 3) were included to the present review. Subsequently, study 2 is referred to as Wood et al. 2015a and study three as Wood et al. 2015b. *Yehuda et al. (2014)* An additional working mechanism of hydrocortisone proposed by the study authors is symptom improvement and reduced drop-out rates by diminishing distress during traumatic memory retrieval. *N* = number of randomised participants, demographic characteristics are reported for participants included in the analyses. In the column *Augmented tf-PT* total number of augmented sessions are listed and in in brackets which sessions in the complete course of therapy were augmented. *Proposed mechanism=* augmentation mechanism proposed by the study authors. *Follow-up Assessments=* post-treatment assessments in the studies. All studies had a baseline assessment before the intervention and at least one post-treatment assessment. Effect sizes in this systematic review were calculated with the first assessment after treatment (T2). Assessment of T2 varied from directly after the last treatment session up to one month after the last treatment session*. Significant Group x time improvement in PTSD symptoms* summarises only the significant between group differences in the listed PTSD assessments. Only the follow-up assessments where significant differences occurred are listed, i.e., if T3 is not mentioned it means that at this time point there were no significant effects found. When several PTSD outcome measurements are listed it is specified in brackets which exact PTSD outcome measurement yielded significant effects. *Prop>Placebo* means that there was a significant greater PTSD symptom reduction in the propranolol group compared to the placebo group*. Placebo>DCS* means that there was a significant smaller symptom reduction in the D-cycloserine group compared to the placebo group. *Side Effects* that are related to the pharmacological augmentation are described in the narrative review part*. We wrote unknown (UNK) when side effects were not reported in the original study. ^1^De Kleine et al. (2012)* if a patient’s PSS-SR scores had dropped below 15 in two subsequent sessions they completed treatment earlier*. ^2^Maples-Keller et al. (2019)* VRET was limited to a minimum of 7 sessions and a maximum of 12 sessions based on improvement in the PSS of 70%. Homework = Indicated when there was between session homework. Side Effects = if there was a difference in reported side effects in the experimental and the placebo group, we listed them in the description of the individual studies. *^3^Yehuda et al. (2015) reported significant group per time interactions that were nonsignificant in a comparison of post-treatment group differences without including initial symptom severity*

**Table 3. Proposed Mechanism of Action of Pharmacological Agents used in the reviewed studies**

| **D-Cycloserine (DCS)** | NMDA receptor agonist enhancing long term potentiation and thus consolidation of the extinction memory. May augment tf-PT by strengthening the novel safety memory trace acquired in therapy (1). |
| --- | --- |
| **Dexamethasone**  **(DEX)** | Glucocorticoid receptor agonist enhancing consolidation of the extinction memory via interaction with the noradrenergic system. May augment tf-PT by strengthening the novel safety memory trace acquired in therapy (2). |
| **Hydrocortisone (HC)** | Synthetic glucocorticoid receptor agonist enhancing consolidation of the extinction memory via interaction with the noradrenergic system. May augment tf-PT by strengthening the novel safety memory trace acquired in therapy (2). |
| **Methylene Blue (MB)** | Methylthioninium chloride promotes neuronal activity by locally enhancing oxygen consumption ability and by interacting with the HPA system, cholinergic system and by impacting the excitability of ion channels, thus enhancing consolidation of the extinction memory. May augment tf-PT by strengthening the novel safety memory trace acquired in therapy (3). |
| **Mifepristone** | Glucocorticoid and progesterone receptor antagonist impairing memory reconsolidation via interaction with the noradrenergic system. May augment tf-PT by weakening the original trauma memory trace activated in therapy (4). |
| **Propranolol** | Beta-noradrenergic receptor antagonist inhibiting noradrenaline stimulated CREB phosphorylation and indirectly disrupting protein synthesis, thus impairing reconsolidation of the trauma memory. May augment tf-PT by weakening the original trauma memory trace activated in therapy (5). |
| **Rapamycin (Sirolimus)** | mTOR inhibitor regulating dendritic protein synthesis, thus impairing reconsolidation of the trauma memory. May augment tf-PT by weakening the original trauma memory trace activated in therapy (6). |

- - - 1. Walker, D. L., Ressler, K. J., Lu, K. T., & Davis, M. (2002). Facilitation of conditioned fear extinction by systemic administration or intra-amygdala infusions of D-cycloserine as assessed with fear-potentiated startle in rats. *Journal of Neuroscience*, *22*(6), 2343-2351
      2. de Quervain, D., Wolf, O. T., & Roozendaal, B. (2019). Glucocorticoid-induced enhancement of extinction—from animal models to clinical trials. *Psychopharmacology*, *236*(1), 183-199.
      3. Wrubel, K. M., Barrett, D., Shumake, J., Johnson, S. E., & Gonzalez-Lima, F. (2007). Methylene blue facilitates the extinction of fear in an animal model of susceptibility to learned helplessness. *Neurobiology of learning and memory*, *87*(2), 209-217.
      4. Flavell, C. R., Gascoyne, R. M., & Lee, J. L. (2020). Postreactivation mifepristone impairs generalization of strongly conditioned contextual fear memories. *Learning & Memory*, *27*(12), 483-487.
      5. Franzellitti, S., Buratti, S., Valbonesi, P., Capuzzo, A., & Fabbri, E. (2011). The β-blocker propranolol affects cAMP-dependent signaling and induces the stress response in Mediterranean mussels, Mytilus galloprovincialis. *Aquatic Toxicology*, *101*(2), 299-308.
      6. Hoeffer, C. A., & Klann, E. (2010). mTOR signaling: at the crossroads of plasticity, memory and disease. *Trends in neurosciences*, *33*(2), 67-75.
